# A qualitative study on Canadian youth’s perspectives of peers who smoke: an opportunity for health promotion

**DOI:** 10.1186/s12889-015-2683-4

**Published:** 2015-12-28

**Authors:** Roberta L. Woodgate, David S. Busolo

**Affiliations:** Faculty of Health Sciences, College of Nursing, University of Manitoba, 89 Curry Place, Winnipeg, MB R3T 2N2 Canada

**Keywords:** Youth, Cigarette Smoking, Peers, Peer influence, Qualitative Research

## Abstract

**Background:**

Peer influence, peer selection, and health risk awareness are factors in smoking among youth. Despite the numerous studies on the social context, social network, and how youth define themselves and their smoking status in relation to tobacco use, qualitative knowledge about the role of smoking within peer relationships from youth themselves is only emerging. In this paper, qualitative findings describing Canadian youth’s perspectives and experiences of smoking within the context of peer relationships are presented.

**Methods:**

To examine youth’s perceptions, a qualitative research study design was used. Seventy-five Canadian youth aged 11–19 years participated in open-ended interviews, focus groups, and photovoice methods. Data analysis involved several levels of analysis consistent with qualitative research.

**Results:**

Youth who smoked were perceived by non-smoking peers as less popular and less socially accepted as represented by the theme: *The coolness (not so cool) factor*. Non-smoking youth felt that peers who smoked strained relationships and forced them to set boundaries and negotiate friendships as denoted by the theme: *Negotiating friendships: Being influenced, but also influencing*. Finally, in the theme of *Making sense of peers who smoke*, youth struggled to understand peers who continued to smoke and why they would start in the first place.

**Conclusions:**

As reinforced in this study, Canadian youth increasingly view smoking as unhealthy and uncool. Moreover, youth report resisting peer influence to smoke and in fact, are now influencing their friends who smoke to quit. The self-empowerment stories of non-smoker youth reinforces the idea that the social meaning of smoking with peers is continuing to change from one where youth accepted and participated in the smoking behaviors of their peers, to an environment where youth’s perceptions of personal health is paramount. Findings from this study could be used to guide health promotion and smoking prevention programs and campaigns for youth.

## Background

The rate of smoking among youth in Canada has been on the decline [1]. In 2012, 11 % (approximately 233,000) of Canadian youth aged 15 to 19 years reported being current smokers which was less than the current smokers (15 %) in 2007 [1]. In spite of this awareness, tobacco use continues to be one of the leading causes of preventable death and disease in Canada [2]. The decline in smoking rates could be partly due to health promotion initiatives that include anti-smoking legislation [3] or because of the changing perspectives towards smoking whereby youth no longer consider smoking to be *cool,* instead preferring healthier lifestyles.

For over two decades, increased attention directed at predicting smoking behavior demonstrates that peer influence, peer selection [4–11], and health risk awareness [12, 13] play important and influential roles. Peer influence and selection that results from different types of friends, peer groups, and crowd affiliations, have a direct effect on the likelihood of youth to smoke or not [4, 5, 8, 14–16]. Peer influence is a covert social process whereby youth modify their attitudes, beliefs, and behaviors to conform to those of their friends [4]. Instead of the more overt approaches like peer pressure, increasingly, research points towards peers influencing and being influenced into smoking behaviors using normative processes [4, 5, 11]. Peer selection is a situation whereby youth choose to interact with other youth who share similar smoking behavior [17]. Peer selection involves affiliation, sharing of group interests, and support for group members [4, 5, 17]. When interests, affiliation or support changes, de-selection is possible. Peers can end their relation with other group members if group interests such as non-smoking change [5].

When it comes to health risk awareness, youth who choose not to smoke often cite health risk awareness as a contributing factor [12, 13, 18]. In a study by Goodall and Appiah [18], Canadian youth who smoked had lesser intentions of smoking in the future after exposure to messages about the health consequences of smoking. Similarly, youth in the United States that took part in the study by Hayes and Plowfield [12] were less likely to start smoking because of health concerns. In a qualitative study by Kulbok et al. [13] that involved 39 Caucasian and African American youth who did not smoke, participants mentioned that they chose not to smoke because of health and addiction concerns. Their decisions were reinforced by parental and peer influence.

Studies on youth smoking support the view that smoking is reinforced within socio-cultural identities where smokers feel socially and emotionally connected to each other and view smoking as a normative practice [19–25]. The research emphasizes that youth acquire information, perceptions, and behavior through social interaction [26]. Focus groups with Greek youth emphasized how smoking was a strong part of their social world [20] while youth in rural China and Sweden shared and gifted cigarettes out of respect and intimacy [21, 27]. In emphasizing the social context of smoking, Poland et al. [22] and Nilsson and Emmelin [27] underline that smoking is a collective social practice rather than an individual behavior. In the study by Nilsson and Emmelin [27], youth who smoked regularly described themselves as belonging to a “smoking community” (p.4).

Overall, the literature reinforces that the mechanisms of peer influence towards smoking appear to be more covert and subtle, reflecting predetermined choices about fitting in, social approval, popularity, and autonomy [4, 25]. However, despite the numerous studies on the social context, social networks, and how youth define themselves and their smoking status in relation to tobacco use [24, 28], qualitative knowledge about the role of smoking within peer relationships from youth themselves is only emerging. Research has shown that adolescents are more likely to adopt smoking behavior that resembles their friends’ behavior [5, 29] because of peer selection and influence. However, little is known about the quality and processes of this influence [4, 29]. As well, we know little about the roles that Canadian non-smoker youth take in their peers smoking behaviors; that is, how Canadian youth describe and define their relationship to their peers and their feelings about and experiences of maintaining these relationships. How smoker and non-smoker youth conceptualize and communicate these messages can help direct health promotion and smoking prevention efforts. Also, by considering the role of youth smoker and non-smoker peer selection and influence, health promoters may tap into the positive interpersonal influence towards smoking prevention particularly by non-smokers. In this paper, we examine Canadian youth’s perspectives and experiences of smoking within the context of peer relationships. The findings emerged from a qualitative study that sought to extend our limited understanding of youth’s perspectives of cancer and cancer prevention including cancer risk [30].

## Methods

Considering we set out to explore how adolescents perceive cancer and cancer risks, we used the qualitative design of ethnography. Adopting this approach helped to ensure a richness of data as youth were afforded the opportunity to present authentic conceptualizations of complex phenomena that cannot be easily uncovered or conveyed through quantitative research [31]. The qualitative research design afforded us the opportunity to understand youth from their frames of reference and experiences of reality.

### Participants and procedures

In a Western Canadian province, 75 youth were recruited from four schools in an urban centre and a rural area. A purposive sampling technique was applied to facilitate the inclusion of male and female youth from diverse backgrounds including both smokers and non-smokers. The sample was considered sufficient as theoretical saturation was achieved.

Data collection mainly took place at the schools the youth attended. Youth took part in two open-ended interviews lasting 60–90 min. For the first interview, an interview guide included questions that were meant to help elicit Canadian youth’s perspectives about cancer and cancer prevention (e.g., What causes cancer?, What are some “things” about cancer that you have heard or been told about?, What type of cancer prevention activities have you taken part in?) was used. The interview guide was developed based on health promotion and cancer prevention literature specific to youth. While there were no direct questions about smoking and peer influence and selection in the guide, the open-ended interview technique provided youth with the opportunity to speak about topics that they considered important [31], which included youth sharing stories about their perspectives and experiences of peers who smoke and do not smoke. Each interview was digitally recorded and transcribed verbatim.

Use of photovoice methods complemented the second interview session. Photovoice is a participatory research method that affords individuals the opportunity to talk about the issues significant to them through photographs and discussion [32–34]. Youth were given a disposable camera and asked to take photographs, over a period of three to five weeks, of people (with permission), objects, places, or events that made them think of cancer and cancer prevention.

During the second interview, youth were asked to describe the photos using the SHOWeD method [35]. For every photo, youth were asked to describe what they saw in the photo, what was happening, and how it related to their lives [35]. Furthermore, questions were asked on why things were happening the way they did and what could be done about it, as well as how the photo could educate others [35]. Youth were also asked follow-up questions based on their initial interview and to comment on any changes since the first interview.

Four focus groups with some of the youth who had participated in the individual interviews were conducted near the end of the study to augment and further explore existing data [36–38]. A total of fourteen youth participated in the focus groups, with each group comprising of three to four youth.

### Analysis

All data emerging from interviews and photographs was organized using *NVivo version 9.0.* [39] Data analysis involved several levels of analysis consistent with qualitative research [40, 41] Inductive coding began with the researchers reading all interview scripts and field notes. The researchers independently isolated patterns referred to as domains. This was followed by organizing domains and identifying attributes for each domain. The last step involved discovering relationships among the domains to create themes. Identified themes were compared and contrasted, then jointly refined. Data from the first set of interviews were compared with the second set of interviews, and themes were refined. Measures to enhance methodological rigor included prolonged engagement with data, careful line-by-line analysis of the transcripts, and detailed memo writing [42]. Sharing the initial interpretations with youth during the second interviews helped to validate the emerging themes.

### Ethical considerations

Throughout the study we strived to ensure that ethical standards were maintained. Prior to data collection, we obtained approval to conduct the study from the Education/Nursing Research Ethics Board at the University of Manitoba. Additionally, we obtained verbal and written parental consent, and assent from all youth participants. All names were replaced with code numbers. Additionally, all photos were handled like any raw data in that any information that could have possibly identified participants or others was removed (digitally altered).

## Results

### Participants’ characteristics

Youth ranged in age from 11 to 19 years old (*M* = 14.5, *SD* = 2.1). Twenty (27 %) youth were boys and fifty-five (73 %) were girls. Forty-seven (63 %) self-identified as European descent, eight (11 %) as Canadian Aboriginal, fourteen (19 %) as other ethnicities (including Asian, African, Jewish, Arabic and Canadian), and six (8 %) participants did not report their ethnic background. Fifty-four (72 %) youth identified themselves as middle-class. Forty-two (56 %) resided in urban areas while thirty-three (44 %) came from rural areas. Four youth in the study identified themselves as smokers and six youth described themselves as past smokers (8 %).

### Themes

Not unexpectedly, smoking was a major focus in the youth’s discussions of cancer and cancer prevention. Youth in this study were well-informed as to how smoking and second-hand smoke affects one’s health (e.g., increased the potential for cancer). Of special importance to youth was how smoking influenced their relationships with peers. Three themes related to peers and smoking emerged in the analysis. These were: 1) the coolness (not so cool) factor; 2) negotiating friendships: being influenced, but also influencing; and 3) making sense of peers who smoke. The themes were similar across the participants’ age, gender, smoking status, and place of residency.

### The coolness (not so cool) factor

When talking about peers who smoked, talk of *coolness* predominated in the youth’s narratives. Both smokers and non-smokers reinforced that youth took up smoking because they wanted to be identified as cool. Being cool was congruent with fitting in or being popular with one’s peers and feeling socially connected as reinforced by the following comment:

Um, I don’t know just cause, like the people I know who do smoke it’s like all their cool older brothers do it so then they want to do it to and be like them, and for some reason they think it will make them popular and stuff. [17-year-old female]

Many people like just do that because they think it’s cool and stuff like that. [13-year-old male]

Despite the fact that youth perceived that others began smoking to be cool, it emerged from the youth that smoking was not cool. A 14-year-old female commented, “So to me it’s just, it’s dumb, it is not cool, and it, it just hurts your body.”

**Fig. 1:**
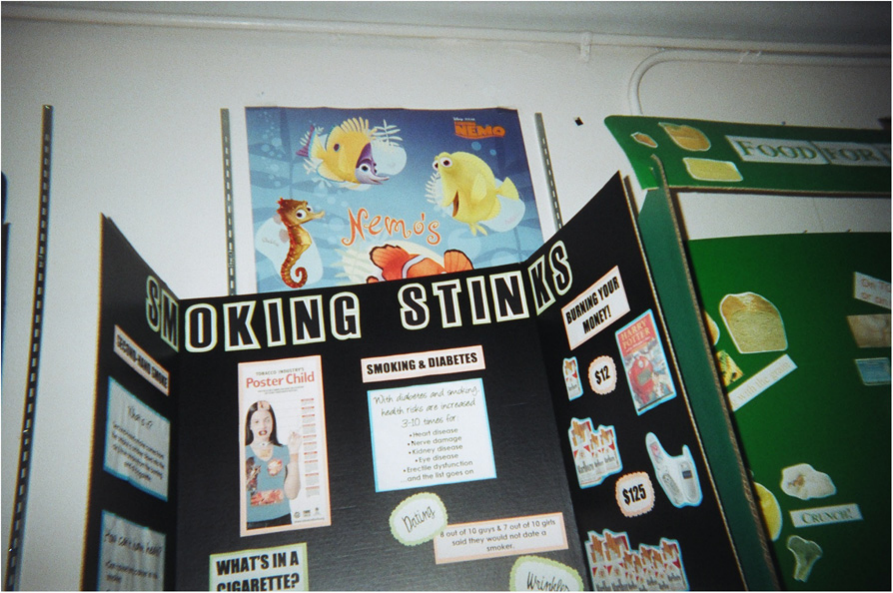
“Smoking is uncool” message

Another 12-year-old female youth who expressed that “some people liked smoking because they think it is cool” felt that smoking was far from cool and in fact was “just so stupid.” To reinforce her point, she took a photograph of a poster that displayed some of the negative impacts of smoking (see Fig. 1).

Even the youth who smoked expressed that smoking was not cool and that the initial coolness factor eventually fades.

Most people they’re like, “It looks cool.” It’s not cool after doing it for five years. You don’t feel like it’s cool. It’s not like “Oh I’m going to go have a smoke.” It’s “I need a cigarette or I’m going to strangle someone!”…The harsh feeling you get in your throat when you wake up in the morning definitely doesn’t feel cool. [16-year-old female]

As opposed to being cool and popular, youth who smoked were perceived as less popular and less likely to be accepted.

Anybody who is caught smoking is pretty much an outcast. Like nobody likes them. Well, because everybody in our school thinks kids that smoke today is just, just the most dumb thing ever, like, you just don’t do that! [14-year-old male]

One youth indicated that smokers at his school were branded as the *smoker kids*; and considered as not fitting in to the school culture. Children who smoked were ostracized and seen as youth who got into trouble.

There’s always a huge crowd of about twenty to thirty people at any time out there smoking…Yeah, they kind of are branded as the smoker kids…You usually don’t want to be one. The people that would talk back to teachers and stuff like that…I would probably classify them as more of a, I guess like a loser almost. Like there’s people at our school that are smokers, but they also do more than just smoke like they do drugs and they drink…and they’re classified as the smoker section…And you’d probably judge them a lot faster based on anything else that they do. [17-year-old female]

### Negotiating friendships: being influenced, but also influencing

Three forms of influence emerged from the narratives. For each type of influence, youth expressed how their friends’ smoking challenged and/or strained their relationships. Smoking also influenced how youth negotiated their friendships with respect to who they befriended and did not befriend.

First, youth were convinced to try smoking by their smoking peers through subtle forms of peer influence. For youth in the study who were smokers, their first attempts at smoking cigarettes occurred when a friend who smoked offered them a cigarette. Their decision to try cigarette smoking in part had to do with having a friend who smoked and “being a friend,” hence they did not mind being swayed by their friends. Youth felt they were persuaded in a friendly manner.

I had a buddy who was smoking at the time too and I was really good friends with him and he told me to try it. I was like “Okay well I don’t know if this is so good for me.” I tried it and I thought it was, uh, fun or whatever and then I kept on doing it and I ended up getting into a serious addiction with it. [16-year-old male]

Several youth started smoking as an act of supporting their friends so they would not have to smoke alone, even though they recognized that their friends influenced them to smoke.

I had a few friends that really pushed on me to start smoking…Maybe they didn’t want to do it alone. I know if I was like doing something that my friend didn’t do, I would probably try and get him, to go along with it so I wouldn’t have to do it by myself…Some company. [19-year-old female]

The second form of influence involved non-smoker youth who resisted pressure and invitations to smoke from peers who smoked. The majority of youth in this study who did not smoke described themselves in terms such as “not much of a risk-taker,” “doing my own thing,” and most important, “not being easily influenced.”

I’ve been to parties before where they’re like, “Oh you want to smoke” and I’m like “No, sorry I don’t smoke.” And like they don’t force on me…because I would say “no” because I have a backbone huh, but um yeah they’ve offered it to me a few times, but I’m not very easily influenced by people. [17-year-old female]

I have friends that smoke. I don’t like smoking myself. They’ll come up to me and ask me if I want to go out for a smoke with them and I’m like, “No I don’t want to participate in that” and people come up to me and be like, “Hey can you buy me some smokes?” I’m like “No I won’t buy you smokes” and they’re like “Can you hold our smokes?” and I’m like “No I won’t even do that.” [17-year-old male]

Finally, a third form of influence that emerged was revealed through stories of youth who did not smoke and were trying to influence their friends who smoked. In short, the non-smoker youth exerted influence over their smoking peers to stop smoking in a form of reverse peer influence.

Lots of my friends quit smoking cause they notice that I don’t smoke…I think it’s actually a pretty good idea that they don’t smoke. Like, I’m happy with myself that I helped them stop smoking because if I helped them stop smoking then they don’t have a chance of getting cancer. [13-year-old male]

Several youth also felt it was important for non-smoking friends to take on a more active and direct role in stopping their friends from smoking.

Like if I were to be smoking, my friends would do whatever they can to stop it or get rid of it…at first they’d do it like all rough and all mean and stuff, but then they’d like do it like, so more soothingly and like they would be more nice about it and like more understanding. And it’s just, it’s just really important to have friends like that cause if you’re by yourself than nobody’s there to stop you and it can get really ugly. [13-year-old female]

**Fig. 2:**
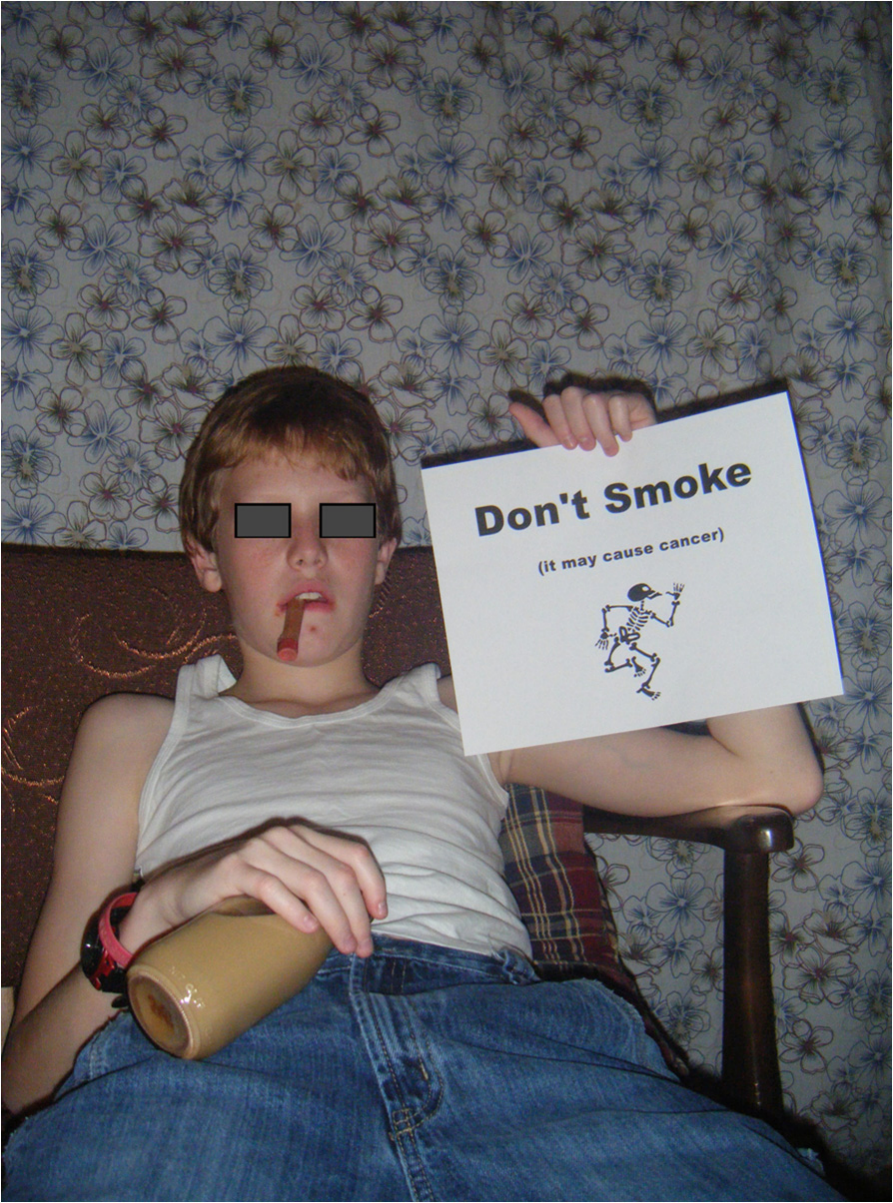
“It is up to you” message

One 12-year-old youth emphasized the importance of empowering friends by encouraging them to be strong and to stand their ground against their smoker peers. Her photograph (Fig. 2) reinforced her point.

**Interviewer:** Okay. So what can you tell me about those posters?

**Youth:** Um, there’s they’re like really powerful I think. Some of them are more powerful than others. Cause this one said attitude is up to you. It takes only one person to change your life. Be smart and ask questions…because if you do not want to smoke, just do not follow what everyone else is doing.

Negotiating friendships emerged as significant when trying to convince peers to quit smoking. This involved establishing firm guidelines, rules, and boundaries for friends who smoked in order to maintain their relationship.

I have a rule that they can’t smoke around me or anywhere near me. If I see them smoking I’ll freaking take the cigarette and like throw it across the street. That’s my rule for all my friends…I know people who smoke but they’re not my friends. I only have like two friends who smoke and they’re not allowed to smoke around me or like talk about it around me. That’s like just my rules… [13-year-old female]

Negotiating friendships also involved tactics such as avoiding peers when they smoked and even ending long-term friendships (de-selection).

I have a friend in my class who is a close friend but she smokes, but I’m never with her when she smokes, so…I would hang out with her, just not when she’s smoking. [15-year-old female]

If two people don’t smoke and one person does smoke they’re not going to want to hang around you anymore. They’re not going to want to be a part of your life. They don’t want to be influenced…and so it probably does affect relationships with like children and just long-time friends. [12-year-old female]

Overall most youth in this study felt that a good friend was someone who would never try to encourage them to smoke. In response to the question of how youth could avoid smoking, one youth expressed,

Um, probably to have really good friends that you know that wouldn’t encourage you not to do those kinds of things. Um, to make friends with people that are, just uh, go to church or something. [13-year-old female]

### Making sense of peers who smoke

Participating youth struggled to make sense of their peers who smoked and why they started smoking in the first place. Some youth viewed smoking as a coping strategy to deal with physical pain and mental distress.

I think it [smoking] hits some like main like nerves or something…and it like takes their pain away, sometimes physical pain…but sometimes for like um just like just because it makes them feel better. [15-year-old female)

**Fig. 3:**
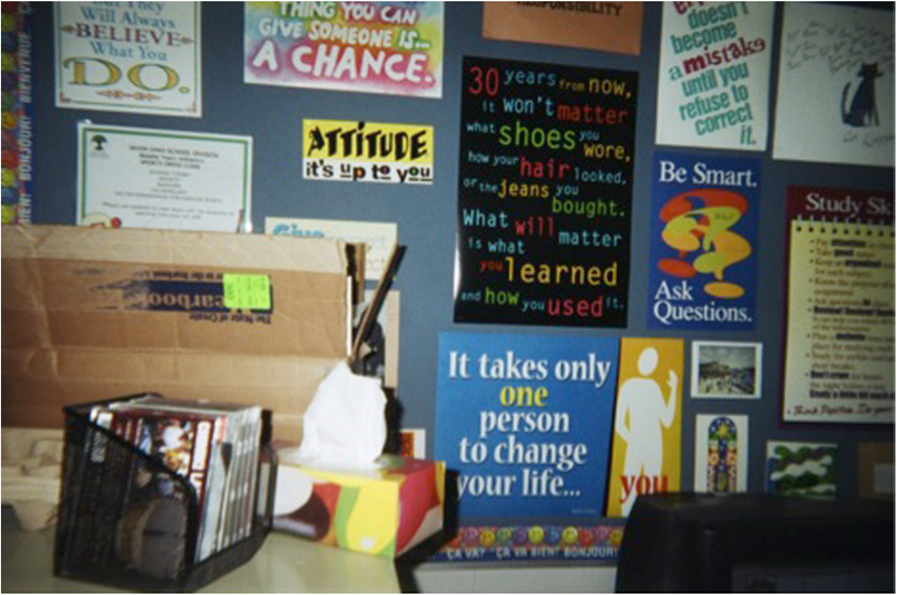
“Getting away from reality” message”

A 13-year-old female felt that smoking was an escape and that people who smoked were unhappy and smoked to get away from their daily lives. In referring to her photograph of a male youth smoking (Fig. 3), she expressed:

I know people who live around me that smoke all the time and I know for a fact that they don’t always look the happiest. Whenever I think of someone smoking I always think of someone who has just that drawn face and…you know something’s going on, but they really in a way can’t help it because they just want to get away from reality and so they’ve gone to this.

**Fig. 4:**
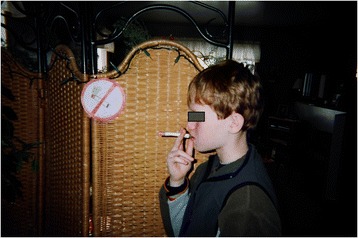
“It makes me feel sad” message

Although youth described smoking as disgusting and careless behavior, they nonetheless expressed empathy and strong emotions for peers who smoked. One 14-year-old female photographed a peer mock smoking (Fig. 4) and said,

I kind of feel hurt and (short pause) yeah I guess sad for that person because I mean he just looks kind of like he’s not really happy. Because I read somewhere that every time you smoke a cigarette you suck five minutes away from your life.

**Fig. 5:**
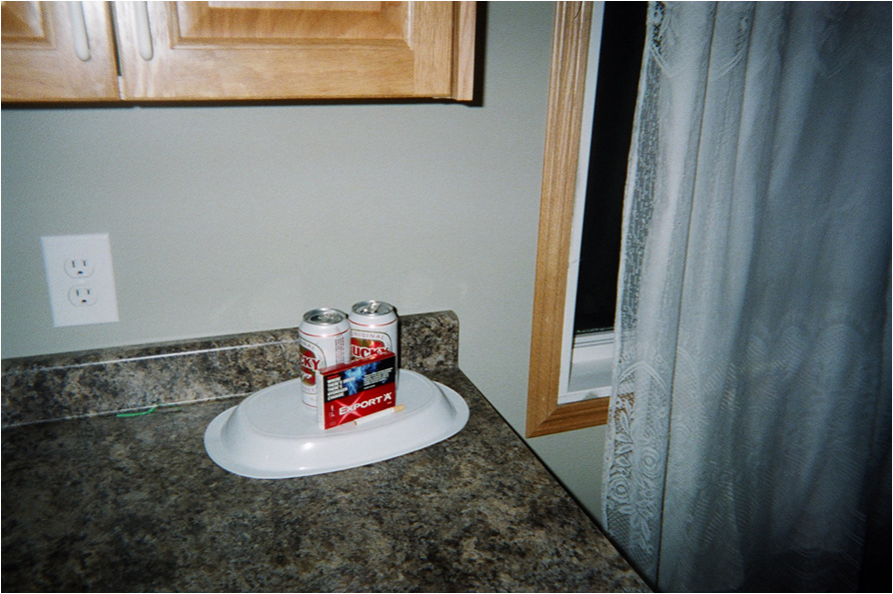
“Taking responsibility for smoking” message

In making sense of smoking by peers, youth nonetheless felt that smoking was both a personal choice and an individual responsibility. In speaking about his photograph of beer cans alongside a package of cigarettes (Fig. 5) a 12-year-old male commented,

Smoking and drinking is bad but everybody has his own responsibility…But I won’t say anything…I don’t say that you can’t do it. Because it’s your own responsibility…You can’t blame anybody else for what you do. So it’s your own decision what you do and what you’re not going to do.

Although recognizing that smoking was a personal choice, youth felt it was important to support youth who smoke by not blaming or judging them.

It’s their choice to smoke and if they want to…and I’m not going to judge them based on what they choose to do because I wouldn’t want them to judge me based on what I choose to do. [17-year-old female]

Youth had empathy for their peers who smoked in spite of acknowledging the negative aspects of smoking. They acknowledged the struggle involved in trying to break the smoking habit.

My friends always say they’re trying to quit and yet they usually don’t. [17-year-old male]

I was watching a commercial and they say that when you don’t have a cigarette then these things in your brain want more and they keep telling you to take a cigarette because they’re used to because they want the nicotine. So then they say eat that nicotine gum so then they’ll be like satisfied. [11-year-old female]

Youth also expressed anger towards tobacco marketing and fear about how easy it was to become addicted to cigarette smoking.

They have smooth and light and rich and different cigarettes like, they have flavours which pisses me off because that just makes it, makes people want to do them more. I mean my cousin he just smoked a cigarillo and now he’s addicted and I’m really mad… I’m scared he’s going to get to be an addict smoker. I mean once you smoke once you’re a smoker. [13-year-old female]

## Discussion

In this qualitative study, youth shared their perspectives about smoking within the context of peer relationships, reinforcing our understandings of smoking and peer relationships [4, 23]. A number of findings emerged from our study that are important to youth educators, especially those involved in smoking prevention and health promotion.

Previous research has revealed that youth engage in smoking because of many complicated factors that include perceived attractiveness and the associations they make between tobacco use and social identity [4, 5, 27]. In spite of these factors, our findings suggest that youth in this study, similar to those youth who participated in the 2012–2013 Youth Smoking Survey, construct themselves in their social environment and within the context of smoking prevention [43]. Our study added more qualitative evidence of youth’s perception of smoking as not being cool. While youth in our study initially spoke of smoking as a way of being cool and popular with peers, it emerged from their deliberations that smoking was not cool. Similarly, only 4 % of youth in the 2006–2007 Youth Smoking Survey felt that smoking was cool [44]. This might suggest that Canadian youth’s self-image may be changing, favouring not smoking and being more assertive (through the use of rule setting as an example) to counteract the peer influence that projects smoking as cool

Our findings were strikingly different from some qualitative studies that examine youth’s smoking behaviors and health risks perceptions. In their study, Tamvakas and Amos [20] found that Greek youth had little awareness of the health risks of second-hand smoke. In fact, youth in their study described smoking as a “social need” and a crucial part of current friendships. Similarly, interviews with Turkish youth revealed that smoking was not seen as a health risk and that the body could recover and heal itself once smokers stopped smoking [45]. Similarly, Swedish youth downplayed assertions that smoking could increase health risks. Health risk denial was mainly as a result of myths that youth used, irrespective of their level of knowledge [27]. In contrast, youth in our study expressed genuine concern about the health-related effects of tobacco use for themselves and for their friends.

Differences in youth’s perception of smoking and its relation to health risk could be a reflection of socio-cultural practices around smoking and the differences in smoking prevention efforts in the different countries. Comparatively, rates of cigarette smoking among youth in European countries like Greece is generally higher than in North American countries like Canada. In 2013, the rates of smoking among youth between ages 15–19 in Greece were 13.74 % among males and 12.98 % among females. These rates were almost twice as high as the rates in Canada. In Canada, 9.97 % of males and 7.56 % of female youth between ages 15 and 17 years smoked cigarettes in 2013 [46]. Over time, Canada has been on the forefront in enacting and implementing smoking prevention legislation [3] and health promotion programs which could have informed perceptions held by youth in our study. Therefore in places where youth continue to perceive smoking as a social need, more health promotion and smoking prevention programs could be carried out, with messages that inform youth about health risks and dispel myths about smoking.

Our research complements socio-cultural and social contextual theories of smoking, which highlight the important role that peers exert in selecting friendships and influencing youth smoking behaviors [4, 7, 8, 10, 23, 47]. Our findings reinforce that peer selection and influence is critical to youth’s smoking behaviors. The few youth in our study with a history of smoking were influenced by those peers who smoked and specifically offered them a cigarette. Similarly in a study in rural China, Hu et al. [21] revealed that among 31 participants (aged 15 and older), sharing cigarettes with peers (and older relations) contributed to individuals starting to smoke. As well, Hu et al. [21] reported that sharing of cigarettes among friends was a major hindrance to smoking cessation. Importantly, our research also revealed how non-smoking youth reduced their interactions with smoking peers and even ended friendships (de-selection) with peers who did not conform to their standards for a smoke-free environment. Similarly Seo and Huang [23] revealed that “isolates” (i.e., youth who do not actively participate in cliques or friendship groups) were more likely to smoke and that their smoking may contribute to social isolation and rejection from non-smoking peer groups.

Our findings demonstrate that by increasingly speaking out through setting boundaries and rules with smoking-peers, youth are protecting themselves and transitioning from a victim into a more empowered role within smoking-peer relationships. It could be suggested that youth’s increased awareness of the harms associated with smoking and second-hand smoke could account for their changing behavior. Youth in our study perceived the act of refraining from smoking and restricting their exposure to smoking peers as a positive and healthy choice. Our findings concur with those that postulate that youth select or choose to relate with other youth of similar smoking status. That is, youth who do not smoke tend to select friends who are non-smokers and also avoid those that smoke [4, 5].

In addition to revealing the complexity of smoking within peer relations, our findings encompassed a strong emotional component. As expressed in other research studies [48], youth described how smoking was perceived as a temporary solution for mental distress, that had the adverse impact of making the smoker youth unhappy . Youth in our study also strongly voiced their concerns for messages that places blame on the smoker for beginning to smoke. A study by DiFranza et al. [49] also supported a strong emotional component by revealing the compulsion to smoke and nicotine dependence as "more intense than wanting, more intrusive and difficult to ignore… if ignored, needing does not abate, it intensifies" (p. 213). As well, Nilsson and Emmelin [27] found that despite being aware that smoking was unacceptable, youth continued to do so to gain control of their emotions and situations. Together, these studies provide a richer description of how emotions shape youth’s experiences of smoking which can be used towards smoking prevention.

Our study results also support the assertion that non-smoking youth could advocate for smoking cessation through their peer selection and peer influence. Non-smoker youth in our study saw themselves as having an influence over their smoker peers, selecting whom to relate with in terms of their smoking status. Youth also perceived a direct connection between their efforts in helping friends quit smoking and cancer prevention which supports research that suggests a positive relationship between peer selection, peer influence and smoking prevention and cessation [4, 5, 23]. The self-empowerment stories of non-smoker youth being the influencer could potentially serve as effective and powerful health promotion messages in youth smoking prevention programs. These programs could be more effective if they are based on current youths’ perception of smoking and smoking prevention and current understanding of peer influence towards smoking and smoking cessation.

There are a number of limitations with our study. First, the study was conducted in Canada where smoking prevention legislation and related health promotion initiatives have been in place for some time. Therefore, youth’s perspectives could have been informed by these factors. Second, the lack of gender differences in the data could be due to the fact that more females than males took part in the study. Third, diversity in ethnic backgrounds and socioeconomic status was not achieved. Fourth, we did not ask youth to quantify the number of their friends who had a history of smoking. Finally and most important, despite trying to recruit both non-smokers and smokers, the majority of youth in our study were non-smokers which may be related to the declining rate of smoking among Canadian youth [1]. While youth shared their perspectives and experiences of smoker and non-smoker peers through qualitative methods, we nonetheless recognize that if more smokers had participated in the study, a different and more complete picture of how non-smokers influence their smoking peers, and vice versa, may have emerged. Future work that accounts for these limitations is warranted to see if the findings in this study are further supported.

## Conclusion

As revealed by the study’s findings, youth increasingly view smoking as unhealthy and uncool. Moreover, Canadian youth report resisting peer influence to smoke and in fact, are now influencing their friends who smoke to quit. The self-empowerment stories of non-smoker youth from Canada reinforce a changing social meaning of smoking with peers from one where youth accepted and participated in the smoking behaviors of their peers and saw smoking as a means to achieve social acceptance, to an environment where youth’s perceptions of personal health override and are more highly valued than “fitting in.” Canadian youth may be forming new self-images based on self-empowerment and rejection of previously acceptable norms of smoking behavior. While youth in our study reported behaviors such as assertive limit-setting and self-protective behaviors, further research is needed on the meanings that youth attribute towards these behaviors, and how we can encourage these health-promotion practices.
